# Exploring the Key Factors of Shared Decision-Making Through an Influential Network Relation Map: The Orthopedic Nurse's Perspective

**DOI:** 10.3389/fmed.2021.762890

**Published:** 2022-01-20

**Authors:** Yanjun Jin, Haiyan Hong, Chao Liu, Ching-Wen Chien, Yen-Ching Chuang, Tao-Hsin Tung

**Affiliations:** ^1^Taizhou Hospital of Zhejiang Province Affiliated to Wenzhou Medical University, Taizhou, China; ^2^Institute for Hospital Management, Tsing Hua University, Shenzhen Campus, Shenzhen, China; ^3^Institute of Public Health & Emergency Management, Taizhou University, Taizhou, China; ^4^Business College, Taizhou University, Taizhou, China; ^5^Evidence-Based Medicine Center, Taizhou Hospital of Zhejiang Province Affiliated to Wenzhou Medical University, Linhai, China

**Keywords:** shared decision-making (SDM), nursing decision, influential network-relation map (INRM), decision-making trial and evaluation laboratory (DEMATEL), multiple criteria decision-making (MCDM)

## Abstract

**Background:**

Few studies have used quantitative methods to explore the key factors affecting shared decision-making (SDM) in nursing decision-making from the perspective of orthopedic nurses.

**Purpose:**

To understand the intercorrelations among shared decision-making questionnaire–nurse (SDM-Q-NUR) factors and identify key factors for clinical nursing care decisions in orthopedics.

**Methods:**

In May 2021, this study investigated the interdependence of the SDM-Q-NUR scale and developed an influential network-relation map (INRM) from the clinical experience of 13 trained orthopedic nurses using the Decision-making Trial and Evaluation Laboratory method.

**Results:**

The INRM results showed that the nine criteria corresponded to three stages: preparation, discussion, and decision. “I helped my patient or patient's family understand all the information” (*C*_5_) and “I wanted to know from my patient or patient's family how they want to be involved in making the nursing care decision” (*C*_2_) are the main key factors for the beginning of nursing decision. In the discussion and decision stages, the corresponding key factors are “I made it clear to my patient or patient's family that a nursing care decision needs to be made” (*C*_1_) and “I asked my patient or patient's family which nursing care option they prefer” (*C*_6_). The result's statistical significance confidence and gap error were 98.106% and 1.894%, respectively.

**Conclusions:**

When making nursing decisions with patients, orthopedic nurses need to have detailed information about how patients are involved in SDM and all relevant information. Nurses should also inform patients and their families regarding the purpose of the discussion, namely, to help one understand the content, advantages, and disadvantages of the nursing care options, and finally, make a decision.

## Introduction

Osteoarthritis is a major disease that causes pain, disability, and socioeconomic costs worldwide (1, 2). Osteoarthritis is characterized by different disease symptoms and treatment options, which means that patients often need to make complicated decisions as part of the medical decision-making process. For example, regarding knee osteoarthritis, the treatment options include lifestyle changes (such as exercise or weight loss), non-steroidal anti-inflammatory drugs, orthoses, and knee surgery (3–6). To make the most appropriate medical decisions, doctors need to communicate with patients and consider their preferences (7–10). This process is called “shared decision-making (SDM).”

The orthopedic nursing process can be mainly divided into two stages. Firstly, the clinical nurse should assess the patient's nutrition and the degree of pain in the affected area during the pre-operative phase. With the help of relevant specialists such as dietitians and anesthesiologists, nutritional support programs and analgesic programs are designed. Then the clinical nurse and the patient are both to select the most appropriate pre-operative care. Secondly, according to the patient's post-operative conditions (including pain, nutrition and psychological factors) in the posyoperative stage, orthopedic nurses and doctors design functional exercise programs, and select appropriate functional exercise programs with patients. In the process of providing orthopedic care, nurses need to fully communicate and discuss with patients to assess the overall situation of patients and choose the most suitable care plan with them.

Identifying the key factors of SDM implementation is essential to improve the quality of medical decision-making (11). Previous studies on the key factors influencing SDM have mainly adopted qualitative methods or literature reviews (12–15). Research in this field mainly discussed the related index system that affects sharing decisions. Some SDM studies have used quantitative methods, such as multiple criteria decision-making (MCDM), to assess treatment selection and key factor problems. For example, Dolan applied the analytic hierarchy process (AHP) to improve communication between patients and medical staff, medical decision-making, and patient care quality (16). Dolan et al. used the AHP approach to assist patients in evaluating priority options for colorectal cancer screening strategies (17). However, the AHP approach assumes that the criteria (factors) are independent, which increases the risk of problematic decision-making (“treating the head when there are headaches, treating the foot when the foot hurts”) (18–20). For this reason, Liu et al. applied the decision-making trial and evaluation laboratory (DEMATEL) to construe the interdependent relationships among criteria and identify the key factors in SDM based on the clinician's perspective (11). These studies highlighted the application value of MCDM methods in SDM and showed that only a few studies had explored the interdependent relationship among the SDM criteria from the clinical experience and perspective of the nurses.

The well-known shared decision-making questionnaire (SDM-Q) scale was developed in 2006 to measure SDM quality between medical staff and patients in terms of medical behaviors (21). In 2010, the patient version of the SDM-Q-9 scale was developed with good internal consistency (α = 0.938) (22). The SDM-Q-9 scale further adopted a 6-point Likert scale [from 0 (completely disagree) to 5 (entirely agree)] and summarized nine measurement items. In 2012, the shared decision-making questionnaire-doctors (SDM-Q-Doc) scale was developed from a physician's perspective (23). The SDM-Q-Doc scale maintained the same wording as SDM-Q-9 and showed good internal consistency (α = 0.88). Currently, the SDM-Q-9 and SDM-Q-Doc have been successfully applied in various studies in the areas of chronic kidney disease (24), otolaryngology (25), adult strabismus (26), and clavicular fracture (27).

Nurses are also one of the main players in the medical process. They have many opportunities to participate in SDM together with clinical patients. Therefore, it is necessary to explore the key factors of SDM in nursing practice based on the nurses' clinical experiences. First, this study adopted the shared decision-making questionnaire–nurse (SDM-Q-NUR) scale as the evaluation criteria model (Table 1), which is based on the integration of items taken from SDM-Q-9 (22), SDM-Q-Doc (23), and PSDM-Q-Parent (28). The Cronbach's Alpha of SDM-Q-NUR scale is 0.938. Second, the DEMATEL method was used to build an influential network-relation map (INRM) from the orthopedic nurses' clinical experiences. Finally, based on the INRM results, the key factors of the SDM-Q-NUR model were identified and discussed.

**Table 1:** The SDM-Q-NUR scale (Cronbach's Alpha = 0.938).

**Criteria**
I made it clear to my patient or patient's family that a nursing care decision needs to be made (*C*_1_)
I wanted to know from my patient or patient's family how they want to be involved in making the nursing care decision (*C*_2_)
I told my patient or patient's family that there are different nursing care options for their medical condition (*C*_3_)
I explained the advantages and disadvantages of the nursing care options to my patient or patient's family (*C*_4_)
I helped my patient or patient's family understand all the information (*C*_5_)
I asked my patient or patient's family which nursing care option they prefer (*C*_6_)
My patient or patient's family and I went over the different nursing care options (*C*_7_)
My patient or patient's family and I selected a nursing care option together (*C*_8_)
My patient or patient's family and I reached an agreement on how to proceed (*C*_9_)

## Materials and Methods

### Study Design and Data Collection

All procedures were performed as per the guidelines of the Institutional Review Board of Taizhou Hospital of Zhejiang Province, affiliated with Wenzhou Medical University (approval number: K20210805) and the tenets of the Declaration of Helsinki. All participant information was anonymously collected. In this study, a structured questionnaire was completed by 13 clinical nurses of the orthopedic department in a case hospital in May 2021.

### The DEMATEL Method

The DEMATEL method was proposed by the Battelle Memorial Institute in 1972. The primary purpose of this method was to illustrate the complex social network structure in the real world (29). The influential network-relation map (INRM) produced by this method is helpful for decision-makers to understand the mutual influence relationship between the criteria and further identify key factors (11, 30). Therefore, this method has been widely used in various fields, such as food insecurity (31), leanness assessment (32), tourism (33), and smart cities (34). For a detailed explanation of this method, refer to previous studies (30, 35, 36). The calculation and a brief description of DEMATEL are as follows.

Step 1: Establishment of an initial influence relation matrix (*A*)

Each orthopedic nurse used a 5-point Likert scale [no impact (0) to very high impact (4)] to evaluate the degree of influence (*i*) to each criterion (*j*) of the SDM-Q-NUR scale. In doing so, an orthopedic nurse direct influence matrix (*E* = [_*e*_*ij*_]*n* × *n*_) was built.

The all-direct influence relation matrices were used to construct an initial influence relation matrix (*A*) by using Equation (1). The degree of average consensus for the matrix (*A*) was tested using Equation (2).


(1) $$\begin{array}{r} A={\left[a_{ij}\right]}_{n\times n}={\left[\frac{1}{u}\overset{u}{\underset{k=1}{\sum }}e_{ij}^{k}\right]}_{n\times n},i,j\in \left\{1,2,\ldots ,n\right\} \end{array}$$

(2) $$\begin{array}{r} \frac{1}{n\left(n-1\right)}\overset{n}{\underset{i=1}{\sum }}\overset{n}{\underset{j=1}{\sum }}\frac{\left|a_{ij}^{k}-a_{ij}^{k-1}\right|}{a_{ij}^{k}}\times 100\% \end{array}$$

Step 2: Establishment of a normalized influence relation matrix (*Z*)

The matrix (*A*) was transformed into a normalized matrix (*Z*) using Equations (3) and (4).


(3) $$\begin{array}{r} Z=A/\varepsilon \end{array}$$

(4) $$\begin{array}{r} \varepsilon =\\ \underset{i,j}{\max}\left\{\max_{i}\sum_{j=1}^{n}a_{ij},\max_{j}\sum_{i=1}^{n}a_{ij}\right\},\;i,j\in \left\{1,2,\ldots ,n\right\} \end{array}$$

Step 3: Deriving the total influence relation matrix (***R***)

The matrix (***R***) is obtained through Equation (5), where *I* is the identity matrix.


(5) $$\begin{array}{r} R=Z^{1}+Z^{2}+\ldots +Z^{h}=Z{\left(I-Z\right)}^{-1},\\ \;when\;\underset{h\to \infty }{\lim}Z^{h}={\left[0\right]}_{n\times n} \end{array}$$

Step 4: Construction of the four influence indices

The matrix (*R*) used Equations (6) and (7) to construct the given (*s*_*i*_) and received (*o*_*i*_) indicators for each criterion.


(6) $$\begin{array}{r} s_{i}={\left(s_{i}\right)}_{n\times 1}={\left[\sum_{j=1}^{n}r_{ij}\right]}_{n\times 1} \end{array}$$

(7) $$\begin{array}{r} o_{i}={\left(o_{i}\right)}_{n\times 1}={\left(o_{j}\right)}_{1\times n}^{{}^{\prime }}={\left[\sum_{i=1}^{n}r_{ij}\right]}_{1\times n}^{{}^{\prime }} \end{array}$$

where the symbol ′ denotes the transpose action.

Then, the given (*s*_*i*_) and received (*o*_*i*_) indicators were combined into the prominence (*s*_*i*_+*o*_*i*_) and relation (*s*_*i*_−*o*_*i*_) indicators for each criterion. The prominence (*s*_*i*_+*o*_*i*_) indicator represents the influence intensity/correlation degree of each criterion in the entire system. The relation (*s*_*i*_−*o*_*i*_) indicator shows the influential nature of each criterion, that is, the cause group (*s*_*i*_−*o*_*i*_>0) and the effect group (*s*_*i*_−*o*_*i*_ < 0).

Step 5: Construction of the INRM and the net influence relation matrix (*D*)

An INRM was constructed based on the prominence (*s*_*i*_+*o*_*i*_) and relation (*s*_*i*_−*o*_*i*_) indicators. The total influence relationship matrix (*R*) determined the net influence relationship for the criteria using Equation (8) and then determined the impact directions between the criteria from the dominance perspective (36).


(8) $$D={\left[d_{ij}\right]}_{n\times n}=\left\{ \begin{array}{l} r_{ij}-r_{ji}>0,\;1\\ r_{ij}-r_{ji}<0,\;0 \end{array}\right.$$

where ***D*** = [_*d*_*ij*_]*n*×*n*_ is the net influence relation matrix. If *d*_*ij*_ = 1, it means that *C*_*i*_ mainly affects *C*_*j*_ (i.e., *C*_*i*_ dominates *C*_*j*_), whereas if *d*_*ij*_ = 0, it means that *C*_*i*_ is mainly influenced by *C*_*j*_ (*C*_*i*_ is dominated by *C*_*j*_). The diagonal elements in the matrix (***D***) are null values because the criteria themselves have only influential relationships and no net influence relationship.

## Results

Table 2 shows that all participants had a bachelor's degree. The ages were mainly <40 years old (69%). Of the participants, 46% were nursing supervisors and their service years ranged from 15 to 20 years (54%).

**Table 2:** The background and characteristics of 13 orthopedic clinical nurses.

**Characteristics**	**Value (%)**
Gender
Male	0 (0%)
Female	13 (100%)
Education
Bachelor	13 (100%)
Master or above	0 (0%)
Age
<40	9 (69%)
≥40	4 (31%)
Professional title
Senior nurse	3 (23%)
Supervisor nurse	6 (46%)
Co-chief nurse	2 (15%)
Chief nurse	2 (15%)
Years of service
Under 10 years	2 (15%)
10–15	2 (15%)
15–20	7 (54%)
20 and above	2 (15%)

An initial influence relation matrix (*A*) (Table 3) from 13 orthopedic nurses' perspectives was constructed using Equation (1). For the matrix (*A*), the statistical significance confidence and gap errors were 98.106% and 1.894%, respectively. Next, the matrix (***A***) used the previously described Equations (3–5) to derive the total influence relation matrix (*R*) (Table 4). Finally, the total influence relation matrix (*R*) was transformed into four influence indices (Table 5) and the net influence relation matrix (*D*) (Table 6) by using Equations (6–8).

**Table 3:** The initial influence matrix.

	***C*_1_**	***C*_2_**	***C*_3_**	***C*_4_**	***C*_5_**	***C*_6_**	***C*_7_**	***C*_8_**	***C*_9_**
*C*_1_	0.000	2.538	2.923	3.154	2.462	2.538	2.846	2.385	2.462
*C*_2_	3.154	0.000	2.462	2.692	2.846	2.615	2.385	2.077	2.308
*C*_3_	2.538	2.231	0.000	2.615	2.538	2.923	2.769	2.846	2.385
*C*_4_	3.000	2.538	2.692	0.000	2.615	2.846	2.462	2.846	2.769
*C*_5_	3.077	3.077	2.692	2.923	0.000	2.923	2.385	2.769	2.385
*C*_6_	2.385	2.077	2.385	2.308	2.154	0.000	2.308	2.692	2.385
*C*_7_	2.615	2.077	2.385	2.769	2.231	3.000	0.000	2.692	2.692
*C*_8_	1.846	1.769	2.385	2.154	1.769	2.538	2.615	0.000	2.769
*C*_9_	1.538	1.615	2.077	2.538	1.846	2.385	2.462	2.769	0.000

*The significant confidence equation is $\frac{1}{n\left(n-1\right)}\overset{n}{\underset{i=1}{\sum }}\overset{n}{\underset{j=1}{\sum }}\frac{\left|a_{ij}^{13}-a_{ij}^{13-1}\right|}{a_{ij}^{13}}\times 100\%=1.894\%<5\%$, i.e., significant confidence is 98.106%*.

**Table 4:** The total influence matrix.

	***C*_1_**	***C*_2_**	***C*_3_**	***C*_4_**	***C*_5_**	***C*_6_**	***C*_7_**	***C*_8_**	***C*_9_**
*C*_1_	0.995	0.998	1.108	1.165	1.020	1.173	1.117	1.140	1.101
*C*_2_	1.090	0.870	1.062	1.118	1.007	1.144	1.070	1.097	1.065
*C*_3_	1.072	0.965	0.968	1.120	1.000	1.161	1.090	1.131	1.074
*C*_4_	1.127	1.010	1.114	1.056	1.038	1.199	1.117	1.171	1.127
*C*_5_	1.154	1.051	1.137	1.196	0.955	1.227	1.138	1.192	1.136
*C*_6_	0.974	0.876	0.973	1.013	0.900	0.945	0.980	1.029	0.981
*C*_7_	1.056	0.942	1.046	1.107	0.971	1.144	0.960	1.107	1.067
*C*_8_	0.913	0.827	0.932	0.965	0.849	1.005	0.951	0.879	0.955
*C*_9_	0.877	0.799	0.896	0.952	0.828	0.972	0.920	0.964	0.819

**Table 5:** The four influence indices.

	***s*_*i*_**	***o*_*i*_**	***s*_*i*_+*o*_*i*_**	***s*_*i*_−*o*_*i*_**	**Cause-effect group**
*C*_1_	9.82	9.26	19.07	0.56	Cause
*C*_2_	9.52	8.34	17.86	1.19	Cause
*C*_3_	9.58	9.24	18.82	0.34	Cause
*C*_4_	9.96	9.69	19.65	0.27	Cause
*C*_5_	10.19	8.57	18.76	1.62	Cause
*C*_6_	8.67	9.97	18.64	−1.30	Effect
*C*_7_	9.40	9.34	18.74	0.06	Cause
*C*_8_	8.28	9.71	17.99	−1.44	Effect
*C*_9_	8.03	9.32	17.35	−1.30	Effect

**Table 6:** The net influence matrix, set and level for each criterion.

	***C*_1_**	***C*_2_**	***C*_3_**	***C*_4_**	***C*_5_**	***C*_6_**	***C*_7_**	***C*_8_**	***C*_9_**	**Net influence set**	**Level**
*C*_1_	–	0	1	1	0	1	1	1	1	*C*_3_, *C*_4_, *C*_6_, *C*_7_, *C*_8_, *C*_9_	6
*C*_2_	1	–	1	1	0	1	1	1	1	*C*_1_, *C*_3_, *C*_4_, *C*_6_, *C*_7_, *C*_8_, *C*_9_	7
*C*_3_	0	0	–	1	0	1	1	1	1	*C*_4_, *C*_6_, *C*_7_, *C*_8_, *C*_9_	5
*C*_4_	0	0	0	–	0	1	1	1	1	*C*_6_, *C*_7_, *C*_8_, *C*_9_	4
*C*_5_	1	1	1	1	–	1	1	1	1	*C*_1_, *C*_2_, *C*_3_, *C*_4_, *C*_6_, *C*_7_, *C*_8_, *C*_9_	8
*C*_6_	0	0	0	0	0	–	0	1	1	*C*_8_, *C*_9_	2
*C*_7_	0	0	0	0	0	1	–	1	1	*C*_6_, *C*_8_, *C*_9_	3
*C*_8_	0	0	0	0	0	0	0	–	0	–	0
*C*_9_	0	0	0	0	0	0	0	1	–	*C*_8_	1

Table 5 shows the influence of the preference/nature of each criterion within the system. Regarding the relation (*s*_*i*_−*o*_*i*_) indicator, “I made it clear to my patient or patient's family that a nursing care decision needs to be made” (*C*_1_), “I wanted to know from my patient or patient's family how they want to be involved in making the nursing care decision” (*C*_2_), “I told my patient or patient's family that there are different nursing care options for their medical condition” (*C*_3_), “I explained the advantages and disadvantages of the nursing care options to my patient or patient's family” (*C*_4_), “I helped my patient or patient's family understand all the information” (*C*_5_), and “My patient or patient's family and I went over the different nursing care options” (*C*_7_) belonged to the cause group of criteria, which are the criteria primarily affecting other criteria in the SDM-Q-NUR scale. In addition, “I asked my patient or patient's family which nursing care option they prefer” (*C*_6_), “My patient or patient's family and I selected a nursing care option together” (*C*_8_), “My patient or patient's family and I reached an agreement on how to proceed” (*C*_9_) belonged to the effect group of criteria, which are the criteria influenced by other criteria in the SDM-Q-NUR scale.

**Figure 1:**
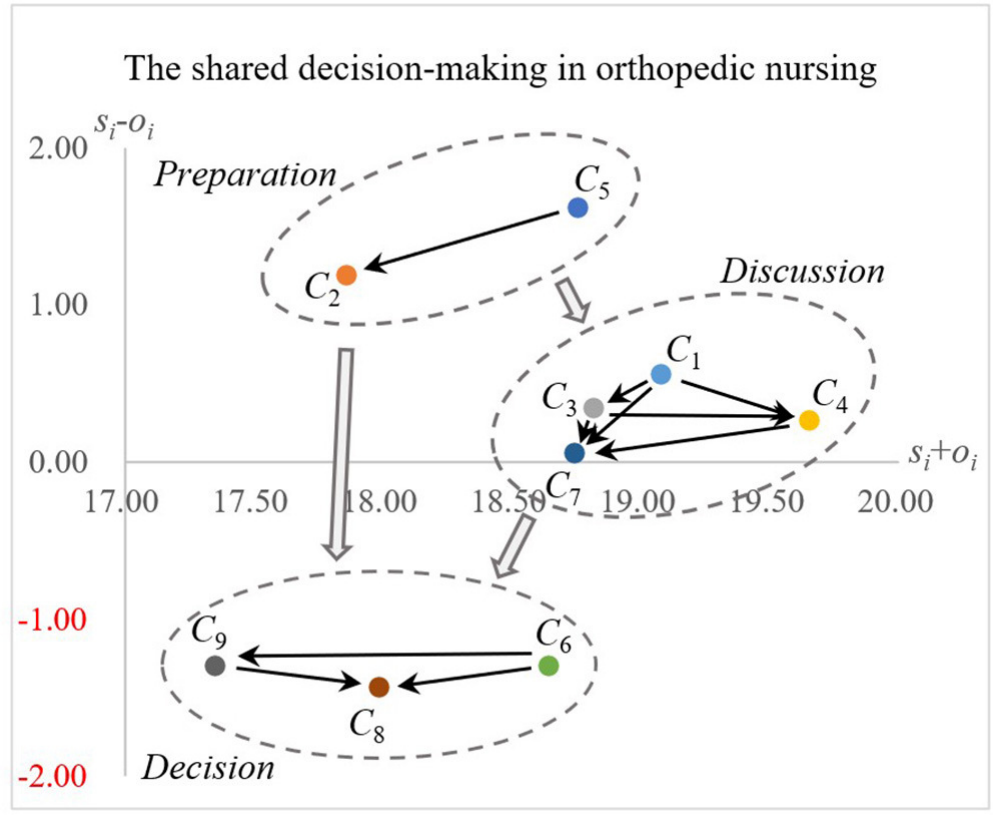
The influential network-relation map of shared decision-making for orthopedic nursing.

Regarding the prominence (*s*_*i*_+*o*_*i*_) indicator, “I explained the advantages and disadvantages of the nursing care options to my patient or patient's family” (*C*_4_), “I made it clear to my patient or patient's family that a nursing care decision needs to be made” (*C*_1_), and “I told my patient or patient's family that there are different nursing care options for their medical condition” (*C*_3_) were identified as the top three, with the highest influence. Finally, the results of Tables 5, 6 were used to construct the INRM (Figure 1).

## Discussions

### Influential Structure Analysis and Clinical Practice

People participate in nursing practice because self-initiating strategies give them a greater sense of independence and empower them in the face of uncertain types of diseases (37). Nurses play a key role in giving patients more knowledge about their health and illness, which may include addressing the clinical agenda, addressing the needs of physical and lifestyle adjustments, improving personal control levels, and changing cultures when necessary (38). Therefore, giving patients the right to participate in choices and make decisions together in comprehensive nursing management can help them to achieve greater control and self-efficacy over their environment (38).

The shared decision-making process includes three interrelated stages: patient participation, discussion and decision-making (39). The INRM (Figure 1) illustrates the interaction structure among the criteria and also shows that the elements of shared decision-making can be divided into three groups: preparation, discussion, and decision in the nursing contents of orthopedic surgery.

#### Preparation

Patient participation in SDM can also be considered as a form of information exchange between clinicians/nurses and patients (39). This relates to “I helped my patient or patient's family understand all the information” (*C*_5_) and “I wanted to know from my patient or patient's family how they want to be involved in making the nursing care decision” (*C*_2_). There is increasing evidence that patients who are better informed and more engaged in their own care are more likely to be knowledgeable, to follow their chosen treatment plans and to have better lifestyle, quality of life and well-being (38). Some studies have also shown that information positively impacts patient participation in decision-making (40–42). Patients' willingness to participate in decision-making depends on many factors, but having relevant information is still one of the key factors (43). Because fear and risk come from people making decisions based on insufficient information (44). Therefore, nurses must provide sufficient and correct information and ensure that patients can understand the available alternatives and make decisions about their care (45). In addition, these factors can be considered key factors in overcoming information asymmetry in shared decision-making. The main reason is that ignorance is prevented when the patient fully understands the key information and then makes the decisions (46). In this phase, nurses should help the patients and their families understand all the orthopedics' care options and provide relevant information for subsequent care discussions, thereby smoothly facilitating the decision-making process. Therefore, before a decision-making discussion, orthopedic nurses should pay attention to ensure all relevant information is provided to the patient and their family.

#### Discussion

The discussion stage is considered to be a two-way form of communication between clinicians/nurses and patients (47). Communication between trusted clinicians/nurses and patients is irreplaceable in a discussion process without prejudice, transparency and comprehensiveness (48). This is why communication between professionals and patients has long been considered very important in providing care and supporting self-management (38). This relates to “I made it clear to my patient or patient's family that a nursing care decision needs to be made” (*C*_1_), “I told my patient or patient's family that there are different nursing care options for their medical condition” (*C*_3_), “I explained the advantages and disadvantages of the nursing care options to my patient or patient's family” (*C*_4_), and “My patient or patient's family and I went over the different nursing care options” (*C*_7_).

At this stage, these factors should have been explained and discussed in the nursing plan. Importantly, nurses need to assist the patients and their families in understanding each care plan and that a nursing care decision must be made. Recognizing and acknowledging that a decision must be made is one of the fundamental factors in shared decision-making (49). The rationale is that when the patient realizes that they must make a decision, the realization will affect their enthusiasm to understand and discuss the nursing plan. Therefore, when discussing orthopedic care plans, the nurse should inform the patients and families that the purpose of the discussion is to assist them in understanding the content, strengths, and weaknesses of the nursing care plan.

#### Decision

The decision phase encourages patients and clinicians/nurses in a way that supports mutual agreement on treatment/care plans (39). This relates to “I asked my patient or patient's family which nursing care option they prefer” (*C*_6_), “My patient or patient's family and I reached an agreement on how to proceed” (*C*_9_), and “My patient or patient's family and I selected a nursing care option together” (*C*_8_). Shared decision-making is essentially based on clinical evidence and further considers the patient's preferences and values for the medical choices (50). Incorporating the patient's values and preferences into the decision-making process is fundamental to shared decision-making (49).

Moreover, the patient's values and preferences are the main factors in deciding the nursing plan at this stage. Therefore, understanding the patient's preferences is paramount. Subsequently, the nurse and the patient decide on the nursing plan together and reach a consensus on the follow-up nursing arrangement. Finally, the orthopedic nursing plan agreed upon by the nurse and the patient is implemented. As such, the nurse and the patient share the consensus, which helps improve the efficiency and quality of the discussion process.

### Methodological Considerations

First, the SDM-Q-NUR scale in this study is based on several SDM-Q scales (i.e., SDM-Q-9, SDM-Q-DOC, and PSDM-Q-PARENT), amended using similar text modification actions. Therefore, the items in the SDM-Q-NUR scale may have a slightly deviated expression for the same meaning. Second, this study mainly discusses the SDM process's influence on orthopedic patients from a nursing perspective. Therefore, this study only considered the nurses' clinical knowledge. The perspectives of doctors were not within the scope of this study. Lastly, the small sample of nurses from the same hospital may bias errors in the results. Therefore, the results should not be extrapolated to hospitals in other regions of China.

### Future Research Directions

First, quantitative research on SDM has mainly focused on clinical interventions, and little research has been conducted to evaluate and improve the SDM process from a management decision perspective. Future research should explore various SDM problems using the MCDM methods with uncertainty theories (e.g., the DEMATEL-based analytic network process or the best-worst method). Second, the variety of applied data mining research based on well-known SDM scales is scarce. In the future, research studies that involve collecting a large number of samples based on several well-known SDM scales (e.g., observer OPTION model or informed decision-making model) and explore the SDM behavior patterns of doctors, nurses, and patients through machine learning methods (e.g., random forest or rough set) should be considered. Finally, based on the same SDM scale, different knowledge viewpoints based on the practical experience of doctors, nurses, and patients may be investigated and analyzed using mixed methods (both qualitative and quantitative methods) in the future. Moreover, the preferences and values of different roles may be analyzed with knowledge differences to further develop the research in SDM.

## Conclusions and Remarks

In this study, the DEMATEL method was proposed to account for the interactions among the criteria in the SDM-Q-NUR scale. The description of these effects may help develop a comprehensive decision-making model. In caring for orthopedic patients, the INRM-based decision model can help nurses understand some of the key factors affecting SDM and thus, improve the decision-making quality of orthopedic nursing. To make nursing decisions with patients, orthopedic nurses need to understand how the patients can participate in SDM and provide all relevant information to the patients. Furthermore, the nurses should inform patients and their families of the purpose for the discussion, assist the patients in understanding the content, advantages, and disadvantages of the nursing choices available, and finally, make a decision.

## Data Availability Statement

The original contributions presented in the study are included in the article/supplementary material, further inquiries can be directed to the corresponding author/s.

## Ethics Statement

All procedures were performed as per the guidelines of the Institutional Review Board of Taizhou Hospital of Zhejiang Province, affiliated with Wenzhou Medical University (approval number: K20210805) and the tenets of the Declaration of Helsinki. All respondents' information was anonymous. The patients/participants provided their written informed consent to participate in this study.

## Author Contributions

YJ and Y-CC conducted the study and drafted the manuscript. HH participated in the design and data collection of the study. CL calculated the results of this study and drew the influence network relation map. T-HT and C-WC conceived the study and participated in its design and coordination. All authors read and approved the final manuscript.

## Conflict of Interest

The authors declare that the research was conducted in the absence of any commercial or financial relationships that could be construed as a potential conflict of interest.

## Publisher's Note

All claims expressed in this article are solely those of the authors and do not necessarily represent those of their affiliated organizations, or those of the publisher, the editors and the reviewers. Any product that may be evaluated in this article, or claim that may be made by its manufacturer, is not guaranteed or endorsed by the publisher.
